# Improved Model‐Data Agreement With Strongly Eddying Ocean Simulations in the Middle‐Late Eocene

**DOI:** 10.1029/2021PA004405

**Published:** 2022-08-17

**Authors:** Peter D. Nooteboom, Michiel Baatsen, Peter K. Bijl, Michael A. Kliphuis, Erik van Sebille, Appy Sluijs, Henk A. Dijkstra, Anna S. von der Heydt

**Affiliations:** ^1^ Department of Physics Institute for Marine and Atmospheric Research Utrecht (IMAU) Utrecht University Utrecht The Netherlands; ^2^ Centre for Complex Systems Studies Utrecht University Utrecht The Netherlands; ^3^ Department of Earth Sciences Utrecht University Utrecht The Netherlands

## Abstract

Model simulations of past climates are increasingly found to compare well with proxy data at a global scale, but regional discrepancies remain. A persistent issue in modeling past greenhouse climates has been the temperature difference between equatorial and (sub‐)polar regions, which is typically much larger in simulations than proxy data suggest. Particularly in the Eocene, multiple temperature proxies suggest extreme warmth in the southwest Pacific Ocean, where model simulations consistently suggest temperate conditions. Here, we present new global ocean model simulations at 0.1° horizontal resolution for the middle‐late Eocene. The eddies in the high‐resolution model affect poleward heat transport and local time‐mean flow in critical regions compared to the noneddying flow in the standard low‐resolution simulations. As a result, the high‐resolution simulations produce higher surface temperatures near Antarctica and lower surface temperatures near the equator compared to the low‐resolution simulations, leading to better correspondence with proxy reconstructions. Crucially, the high‐resolution simulations are also much more consistent with biogeographic patterns in endemic‐Antarctic and low‐latitude‐derived plankton, and thus resolve the long‐standing discrepancy of warm subpolar ocean temperatures and isolating polar gyre circulation. The results imply that strongly eddying model simulations are required to reconcile discrepancies between regional proxy data and models, and demonstrate the importance of accurate regional paleobathymetry for proxy‐model comparisons.

## Key Points

- Eddying ocean simulations provide a profoundly different local flow compared to noneddying simulations
- Heat transport is enhanced in eddying simulations leading to reduced equator‐to‐pole sea surface temperature gradients
- Eddying simulations reduce model‐data mismatches for sea surface temperature and ocean flow

## 1 Introduction

Model‐data comparisons for warm periods in the geological past can be used to test the performance of climate models under greenhouse conditions (Braconnot et al., 2012; Cramwinckel et al., 2018; Dowsett et al., 2013; Hutchinson et al., 2021; Kennedy‐Asser et al., 2020; Liu et al., 2009; Lunt et al., 2021; Schmidt et al., 2014; Tabor et al., 2016; Tierney et al., 2020; Zhu et al., 2020). Some fully coupled climate models using state‐of‐the‐art Eocene geographic boundary conditions (Baatsen et al., 2016) and greenhouse gas forcing simulate climates that correspond well to reconstructions of tropical sea surface temperature (SST) and deep ocean temperature (Cramwinckel et al., 2018). However, in such simulations, these models regionally simulate much cooler conditions in extratropical regions than proxy data suggest, particularly in the southwest Pacific (Baatsen et al., 2020; Cramwinckel et al., 2018; Huber & Caballero, 2011; Lunt et al., 2012, 2021). Consequently, depending on the radiative forcing, models either produce SSTs near the equator that are higher than proxy data indicate or SSTs at mid‐to‐high latitudes that are much lower than proxy reconstructions, leading to stronger meridional SST gradients.

One challenge in paleoclimate model‐data comparisons is the scale difference between proxies and models. The proxies capture a regional environment and effects of small‐scale regional setting (e.g., geography, bathymetry, and oceanography), while general circulation models have difficulties capturing regional climate correctly due to the coarse resolution that is typically used (1° horizontally or coarser for the ocean) (Dowsett et al., 2013; Eyring et al., 2019; Harrison et al., 2016; Kennedy‐Asser et al., 2020; Nooteboom et al., 2020; Tabor et al., 2016). The quality of ocean models improves considerably at a higher horizontal resolution (0.1°) (Dong et al., 2014; Griffies et al., 2015; Hewitt et al., 2016; McClean et al., 2006; Müller et al., 2019; Sun et al., 2019; Viebahn et al., 2016), especially their regional flow (Delworth et al., 2012; Marzocchi et al., 2015; Nooteboom et al., 2020). This is not only due to higher level of detail, but also because of the smaller scale interactions resolved (including mesoscale eddies of 10–30‐km size) that influence the large‐scale flow properties (Porta Mana & Zanna, 2014) and increase the importance of the local setting (i.e., the paleogeography and bathymetry) in the resulting regional ocean flow.

Biogeographic patterns of microplankton (e.g., dinoflagellate cysts; dinocysts) in Southern Ocean marine sediments have been used as tracer of past surface oceanography (Huber et al., 2004). For instance, Eocene sediments deposited near Antarctica contain dinocyst species that are endemic to circum‐Antarctic locations (Bijl et al., 2011). Hence, Southern Ocean regions with many of these endemic species, as opposed to those with abundant cosmopolitan species, must be oceanographically connected. This implies that these biogeographic patterns of dinocysts provide a direct proxy of the flow direction itself (Bijl et al., 2011). So far, climate models were broadly able to match the circulation patterns deduced from microplankton endemism in the Southern Ocean, sometimes after adaptations of the model paleobathymetry (Bijl et al., 2013; Houben et al., 2019; Huber et al., 2004) or details of the configuration of critical Southern Ocean gateways (Sijp et al., 2016). However, these model simulations cannot explain the occurrence or absence of endemic dinocysts at some sites. In addition, state‐of‐the‐art fully coupled climate model simulations did come close to the proxy‐based warmth in the southwest Pacific Ocean, but this required a flow through the Tasmanian Gateway which was incompatible with microplankton‐based evidence of surface ocean flow (Cramwinckel et al., 2020; Stickley et al., 2004). Consequently, no model simulation exists that can reconcile southwest Pacific Ocean warmth with ocean flow that is compatible with the plankton records (Baatsen et al., 2020).

Model‐data mismatches of ocean circulation and climate occur from the early to late Eocene (Bijl et al., 2013; Cramwinckel et al., 2018, 2020; Houben et al., 2019; Inglis et al., 2015; Stickley et al., 2004). Here, we show that high‐resolution ocean model simulations partly solve this mismatch during the middle‐late Eocene, using sinking Lagrangian particles to represent biogeographic patterns of microplankton in the ocean model simulations (Huber et al., 2004; Nooteboom et al., 2019). We present the first simulations of a global eddying Eocene Ocean model with a 0.1° horizontal resolution (HR2 and HR4; Table 1). These simulations are initialized and forced with atmospheric fields from an equilibrium state of a coarser (1°) resolution model with a fully coupled ocean and atmosphere (LR2 and LR4; Table 1; Baatsen et al., 2020). Hence, the high‐resolution and low‐resolution simulations have a similar atmospheric forcing and bathymetry. The new high‐resolution simulations are run for a few decades (42 and 27 years for HR2 and HR4, respectively), sufficient for the upper‐ocean circulation to equilibrate. We focus on the middle‐late Eocene in this paper, because of the availability of low‐resolution simulations (Baatsen et al., 2020) and substantial field data of ocean circulation and climate during this time period.

**Table 1:** The Ocean Model Simulations of the Middle‐Late Eocene (38 Ma) in This Paper

Run	Resolution	Layers	Type	Forcing^a^	Years run
LR2^a^	1°	60	Fully coupled with atmosphere (CESM)	2× preindustrial CO_2_	3,000
LR4^a^	1°	60	Fully coupled with atmosphere (CESM)	4× preindustrial CO_2_	4,000
HR2	0.1°	42	Ocean only (POP), forced by LR2 atmosphere	2× preindustrial CO_2_	42
HR4	0.1°	42	Ocean only (POP), forced by LR4 atmosphere	4× preindustrial CO_2_	27

^a^ From Baatsen et al. (2020).

## 2 Materials and Methods

### 2.1 Data

We used two data sets in this paper. The first includes the SST proxies from $U_{37}^{k}$, ${\text{TEX}}_{86}^{H}$, Mg/Ca, Δ_47_ and *δ* ^18^O, which are described in detail in Baatsen et al. (2020). Proxy‐based SST reconstructions come with uncertainties, limitations, and biases (Hollis et al., 2019), related to the depth, or season they represent. The second data set are sediment samples with dinocysts from Bijl et al. (2011), combined with the samples described in Bijl et al. (2021), Cramwinckel et al. (2020), and Houben et al. (2019). We averaged dinocyst abundance of Endemic‐Antarctic, cosmopolitan and low‐latitude‐derived for the respective time slices.

### 2.2 Model Setup

We used the Parallel Ocean Program (POP; Smith et al., 2010) to perform eddying ocean model simulations for the middle‐late Eocene (38 Ma). To derive the forcing of this model, we made use of the fully coupled (ocean and atmosphere) simulations with the Community Earth System Model v1.0.5 (CESM) from Baatsen et al. (2020), with a noneddying ocean. We used both CESM simulations with 2× preindustrial atmospheric CO_2_ (LR2) and 4× preindustrial CO_2_ (LR4) configuration. The setup of POP is similar to that in Viebahn et al. (2016), den Toom et al. (2014), McClean et al. (2011), and Kirtman et al. (2012) with 42 vertical layers (Figure S1 in Supporting Information S1). The K‐profile parameterization was used for vertical mixing.

The high‐resolution POP is forced at the surface by a fixed atmosphere of the CESM simulation. To construct the surface forcing, we interpolated the average (over the last 50 model years of LR2 and LR4) SST, sea surface salinity (SSS), and wind stress (zonal and meridional) of the CESM simulation for every month of the year (such that a seasonal cycle is included in the surface forcing and determines most of the variability). These SST and SSS fields were used as restoring boundary conditions at the surface.

Restoring boundary conditions imply that POP is “pushed” toward the SST and SSS output of the CESM at the surface with a specific time scale (30 and 10^20^ days, respectively). SSS is by approximation free to evolve due to the long restoring time scale, which implies that we use mixed boundary conditions in this paper. Typically, restoring boundary conditions allow for less SST variability compared to a configuration with a fully coupled atmosphere, since SST is pushed toward this fixed solution. The bathymetry that CESM uses was interpolated linearly on the high‐resolution grid that POP uses, making both bathymetries similar (see the code at Nooteboom (2022a)).

In order to investigate the sensitivity of simulations to model resolution, we choose a setup of the eddying model configurations which is as similar as possible to the noneddying model configurations. This means that the eddying simulations do not use a bathymetry which is more detailed compared to the noneddying simulations. The addition of a detailed bathymetry increases the bottom roughness, and hence has implications for the flow (Sauermilch et al., 2021).

For initialization of the eddying model, the three‐dimensional ocean output at the end of the CESM simulations (LR2 and LR4) is interpolated to the higher resolution grid that POP (HR2 and HR4) uses. We simulated 42 and 27 years in total for HR2 and HR4, respectively. Since we investigate the response of the simulations to an increase in horizontal resolution, the same five model years of both HR2 and HR4 are used in most analyses in this paper: years 23–27. For the same analyses of the low‐resolution simulations (LR2 and LR4), we used the last 5 years of these simulations.

Using this setup of POP, we can investigate the sensitivity of simulations to the studied resolution difference only, because the model is forced by the same atmosphere and their geographic boundary conditions are based on the same reconstruction of Baatsen et al. (2016), and the three‐dimensional eddying ocean is initialized by the equilibrated output of the CESM. As a result, the atmosphere is representative of the middle‐late Eocene climate, but does not respond to changes in the ocean. We hence cannot investigate the effect of atmospheric feedbacks on the results (Arzel et al., 2011; den Toom et al., 2012; Rahmstorf & Willebrand, 1995; Zhang et al., 2010).

Similar model setups of POP have been used in studies of the present‐day, to study the response of overturning changes after surface freshwater perturbations (den Toom et al., 2014; Weijer et al., 2012), multidecadal variability (Le Bars et al., 2016) and sensitivity of the ocean heat transport to Southern Ocean gateway changes (Viebahn et al., 2016). Most of these studies show that ∼100 years of spin‐up is long enough for these present‐day uncoupled eddying simulations to be in equilibrium in the upper to mid ocean, which is longer than the eddying simulations presented in this paper. In this paper, we study the response of the simulation to an increase in horizontal model resolution. Hence, we assume that the noneddying simulations, where the eddying simulations are based on, are in equilibrium and representative of the studied time period. Since we use Lagrangian particles, only the upper ocean should be in equilibrium, which is the case after ∼15 years (Maltrud et al., 2010). The uncoupled model setup may not represent ocean heat transport well at long time scales, since it implicitly assumes an atmosphere with infinite heat capacity, and the atmosphere of the noneddying simulations may not be compatible with the SST distribution in the eddying simulations (Huber et al., 2003).

The model setup is suited to study the effects of model resolution on Eocene Ocean flows, but it is not suitable to study dynamics which involve atmospheric coupling, such as the El Niño Southern Oscillation. The model setup can best be used to investigate the upper‐ocean circulation, as the deep ocean is not in equilibrium yet. Therefore, we can only use this setup to obtain a transient response of the deep meridional overturning, not its equilibrium.

Since the restoring boundary conditions are fixed in the uncoupled POP simulations, the SST distribution is determined by a combination of these boundary conditions and internal heat transport within the ocean. Inconsistencies may arise between the direct ocean heat transport in the model and the implied ocean heat transport from the atmosphere, since the atmosphere is fixed (Huber et al., 2003), but cannot be avoided. We assume that this inconsistency is small in this paper compared to other studies that use OGCMs to test the sensitivity of ocean heat transport on gateway changes, where the deep ocean circulation typically changes more compared to the sensitivity to model resolution tested in this paper. The high model resolution itself does influence the thermocline depth (Small et al., 2014), however, and hence, the distribution of heat in the upper layers of the ocean.

The 38 Ma time slice paleogeography was chosen here, because it is also used by Baatsen et al. (2020), which our eddying simulations are based on. Moreover, suitable field data are provided for the middle‐late Eocene Ocean circulation and climate, which allow us to properly test the effect of model resolution on the model‐data agreement. HR2 is forced by an atmosphere with a CO_2_ concentration that is representative for the 38 Ma time slice. HR4 is warmer, and since paleogeography did not change much throughout the Eocene (Lunt et al., 2021), HR4 is more representative of an earlier period in the Eocene (40–45 Ma) when geography was still similar (Baatsen et al., 2020).

### 2.3 Sinking Lagrangian Particles

**Figure 1:**
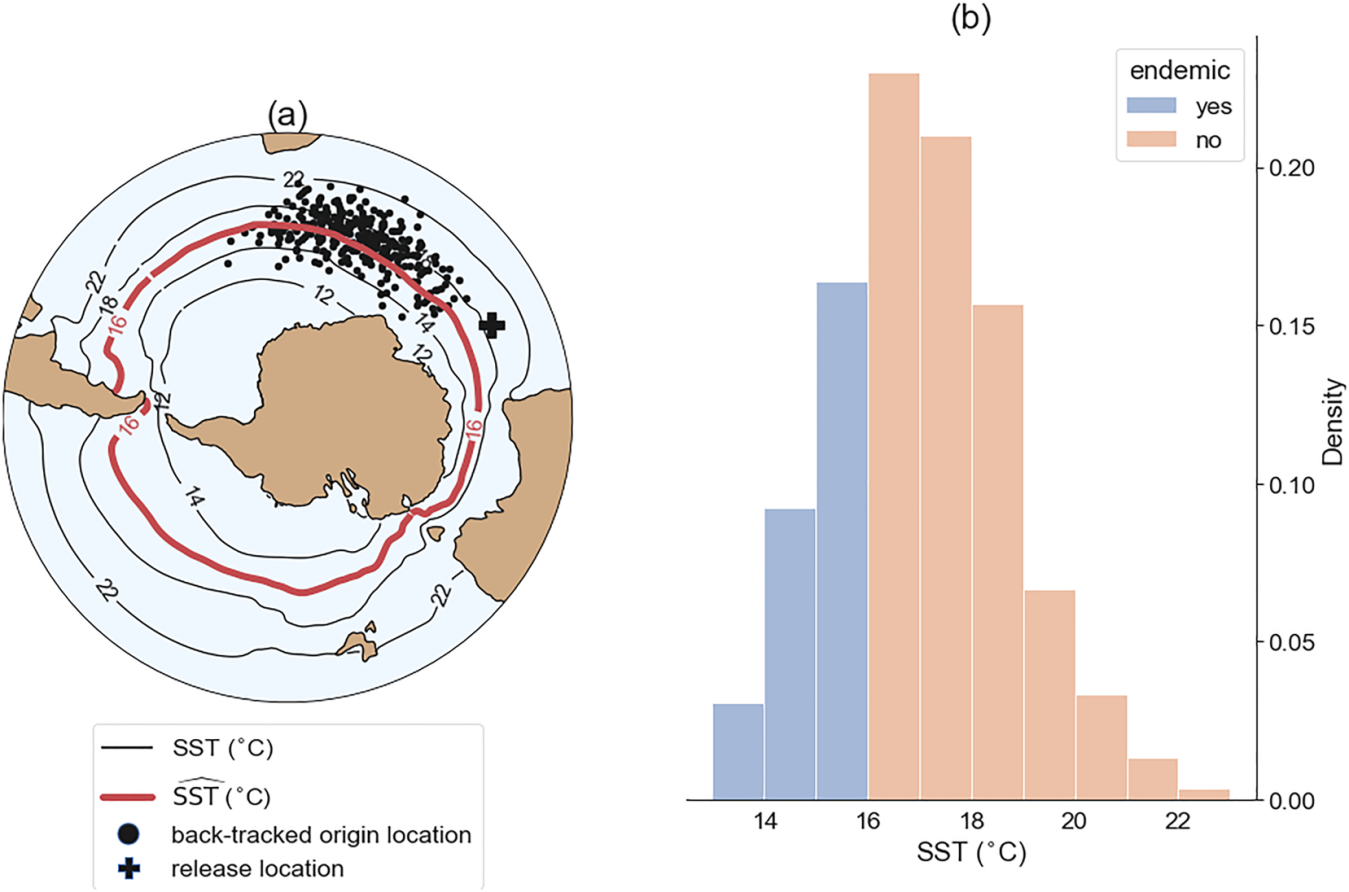
Illustration of the modeled dinocyst endemism near Antarctica. (a) Virtual particles are released at the bottom release location and tracked back in time with some sinking speed to determine their surface origin location. If the sea surface temperature (SST) at the back‐tracked origin location is lower than the threshold SST ($\hat{SST}=16{}^{\circ}C$ in this illustration), it is assumed to originate close to Antarctica; hence, it is flagged as endemic. (b) A histogram of SSTs at the surface origin locations.

To quantify sedimentary dinocyst endemism in the model, we applied a similar backtracking analysis of virtual sinking Lagrangian particles as in Nooteboom et al. (2019) (Figure 1). We released these particles at the ocean bottom and tracked them back in time while sinking and being advected by the three‐dimensional flow from POP, until they reached 10‐m depth. We released particles on a 2° × 1° grid of locations between 32°S and 80°S every day for a year and waited until all of the particles reached the near‐surface (i.e., 17,520 particles in total). This analysis requires a higher than monthly temporal resolution of model output (Nooteboom et al., 2020; Qin et al., 2014). Therefore, we used daily fields for the years 35–42 (HR2) and years 20–27 (HR4) to perform this backtracking analysis.

We used a particle sinking speed of 6 m day^−1^ for the Lagrangian particles in this paper. This represents a low sinking speed for single dinocysts (Anderson et al., 1985). We choose this low sinking speed, because it is considered as a lower bound of the realistic sinking speeds where most lateral transport occurs, which makes it easier to explain low abundances of dinocyst species. However, this sinking speed could in reality be different due to, e.g., aggregation with other particles. We also applied a sinking speed of 25 m day^−1^ (see Figure S2 in Supporting Information S1), which represent small aggregates (Nooteboom et al., 2019). The main conclusions on the model‐data comparison do not change if 25 m day^−1^ instead of 6 m day^−1^ sinking speed is used.

This paper follows previous assumptions (Huber et al., 2004) that the biogeographic distribution of Antarctic endemism was temperature‐controlled. Bijl et al. (2011) recognized that the biogeographic distribution of Antarctic‐endemic dinocysts followed the broad pattern of gyral ocean circulation in the Eocene, in both the South Pacific and the South Atlantic. This implied dominance of endemism in the northward flowing western boundary currents in the southwest Pacific and Atlantic. This paper assumes that presence of Antarctic‐endemic dinocysts outside “cold” regions must have been the result of lateral transport through ocean currents (following the approach of Nooteboom et al. (2019)). Although this is not in disagreement with Bijl et al. (2011), it assigns a larger role to lateral transport instead and a tighter temperature tolerance to endemic dinocysts than in Bijl et al. (2011).

The percentage of dinocyst endemism in the model is determined by the percentage of particles that originated from an environment with a temperature below $\hat{SST}$ when it reaches the surface (which must be close to Antarctica; similar approach as in Huber et al. (2004)). The percentage of modeled dinocyst endemism is not expected to compare well with the percentage of measured endemic dinocyst, because this match is also sensitive to the species‐specific susceptibility of dissolution during the sinking journey and their productivity at the ocean surface (Nooteboom et al., 2019). Therefore, we compare whether any endemic species occur in sites (0% or >0%) between model and data instead of the exact percentage.

We assume that the sinking Lagrangian particles are not greatly influenced by the fact that the deep circulation is not in full equilibrium yet in the eddying simulations. Most of the lateral particle displacement occurs near the surface which is in equilibrium and where the currents are the strongest. Moreover, the eddying simulations are initialized with output from the noneddying simulations, which are in reasonable equilibrium. The mechanistic development of the flow, given the heat and salt distribution from the initialization, occurs in a few years (see also Figures 4c–4h). Hereafter, the flow changes slowly and may equilibrate after ∼1,000 years due to the flow response to changing density distributions. The assumption that sinking Lagrangian particles are not greatly affected by the deep ocean equilibration is supported by the results that use sinking Lagrangian particles in HR2 and HR4: These results are similar, even though the deep ocean circulation is different in HR2 and HR4.

## 3 Effect of Model Resolution on Eocene Flow

**Figure 2:**
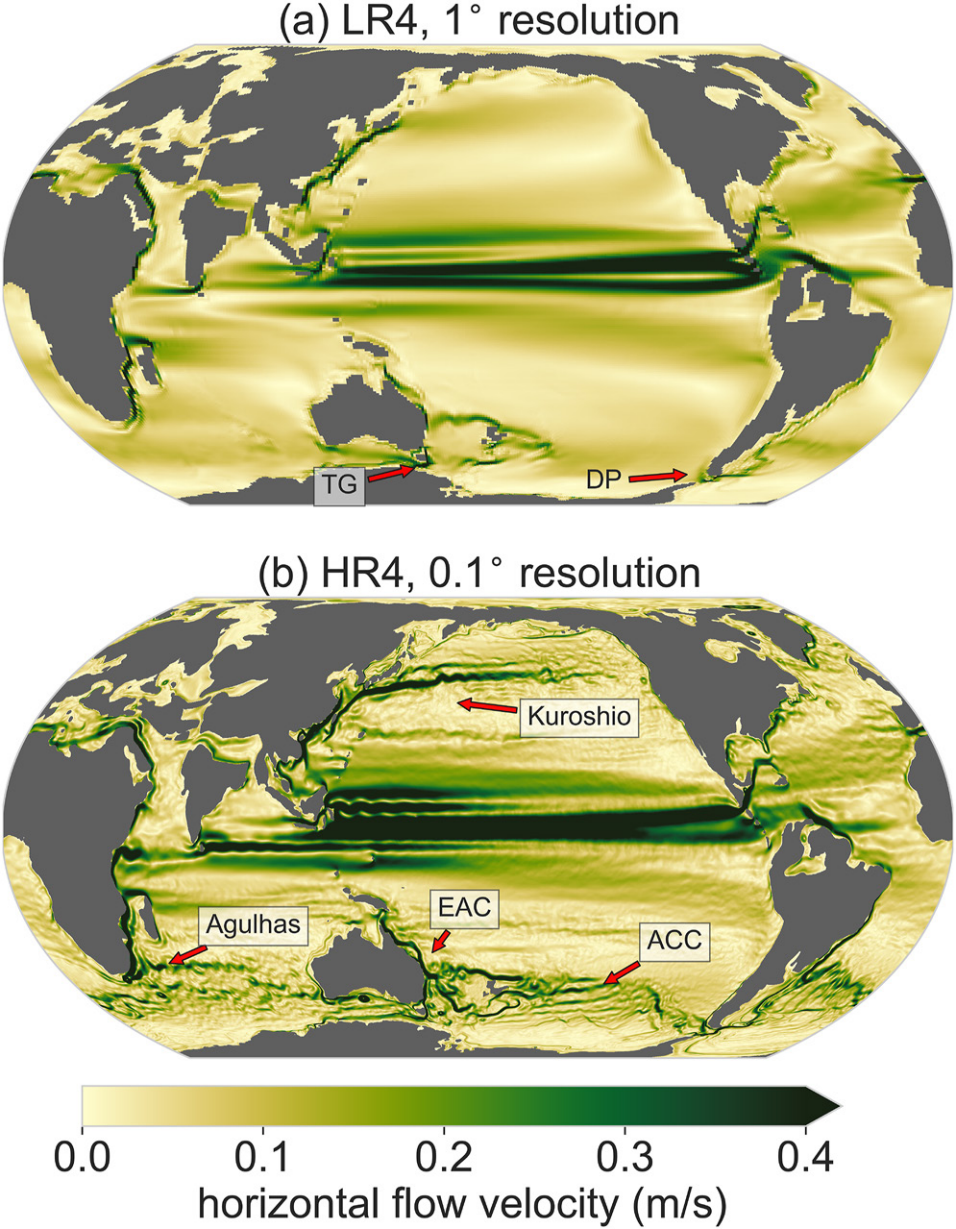
Magnitude of the time‐mean surface horizontal flow velocity in the model of (a) 1° (mean over years 3995–4000) and (b) 0.1° horizontal resolution (mean over years 23–27). Both with 4× preindustrial atmospheric CO_2_ (LR4 and HR4). See Figure S4 in Supporting Information S1 for the barotropic stream functions in all configurations. The Drake Passage (DP), Tasman Gateway (TG), East Australian Current (EAC), Kuroshio current, and proto‐Antarctic Circumpolar Current (ACC) are labeled.

The resulting ocean circulation is different between the eddying and noneddying configurations (Figure 2). In the eddying simulations, the time‐mean flow strength has a higher spatial variability, the bathymetry has a larger influence on the flow strength and direction (especially in the Southern Ocean; see Figure S3 in Supporting Information S1 for the bathymetry), and local scale features are much more pronounced, compared to the low‐resolution model. All western boundary and equatorial currents increase in strength, except in the North Atlantic. The spatial structure and separation locations of the western boundary currents are also shifted. For instance, the eastward Agulhas separation (near South‐Africa) is only present in the eddying simulations (it retroflects more eastward compared to the present‐day). Moreover, east of Australia, the East Australia Current (EAC) extends further southeastwards in the eddying compared to the noneddying simulation, while there is a narrow but strong northward current east of Tasmania that is not present in the low‐resolution simulations.

The EAC flow provides an example of the stronger influence of the paleobathymetry on the flow in HR4 compared to LR4, even though the bathymetry is the same in both configurations. Eddies are responsible for the downward transfer of momentum input at the ocean surface by winds that is eventually balanced by bottom form stresses (Munday et al., 2015). As a consequence, the flow is strongly determined by isobaths (i.e., lines of constant bathymetry; Marshall, 1994; Rintoul, 2018). Hence, the bathymetry has a much larger influence on the flow if the ocean is eddying (in HR4 and HR2) than if it is not (LR2 and LR4). In HR4, the EAC is steered further southeastward than in LR4 along the submerged continental block of Lord Howe Rise (see Figure S3 in Supporting Information S1 for the bathymetry). Moreover, jets like the EAC have a narrower structure in the eddying flow, due to interactions between eddies and the time‐mean flow (Waterman et al., 2011), which has profound impacts on the regional oceanography.

### 3.1 Model‐Data Comparison: Plankton Biogeography

**Figure 3:**
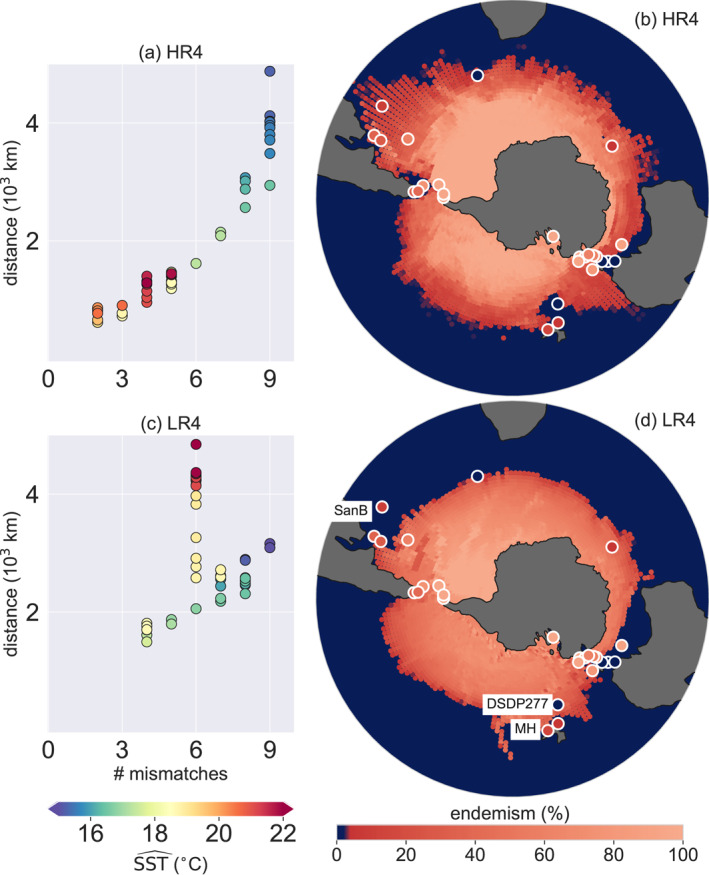
Model‐data comparison: Antarctic endemism of sedimentary dinocysts in configurations HR4 and LR4. Both HR4 and LR4 are compared to 27 sites in total. The model dinocyst endemism at the ocean bottom is determined by the percentage of virtual particles that started sinking (with 6 m day^−1^ sinking speed) in a surface environment with temperature below $\hat{SST}$ (see Figure 1 for an illustration). (a, c) Model‐data fit for HR4 and LR4, respectively, for different values of $\hat{SST}$ (given by the dot colors). Model and data compare better if the following two measures of fit are lower: (1) the number of sites with a point‐to‐point model‐data mismatch in terms of endemic dinocyst species occurrence and (2) shortest cumulative distance of these sites to a location in the model that does match in terms of endemic dinocyst occurrence (i.e., ∑_*i*_ *D*_*i*_, where *D*_*i*_ is the distance between a site *i* and a location in the model that does match with site *i* in terms of the endemic dinocyst occurrence). (b, d) Model‐data comparison of dinocyst endemism at the $\hat{SST}$ value that minimizes the measures of fit in (a) and (c). The sedimentary endemism of the data is the percentage of measured endemic species at the site (Bijl et al., 2011), representative of 41‐39 Ma. Labeled sites are named in the main text.

The new Eocene Ocean model velocity fields enable the use of sinking Lagrangian particles (Nooteboom et al., 2020) to reveal biogeographic provinces of endemic microplankton in the Eocene Southern Ocean. In this way, we can test how representative the modeled flow is compared with the reconstructed ocean flow from sediment records. In this approach, it is determined where sedimentary particles originated from at the ocean surface, while taking into account how the particles were advected by ocean currents during their sinking journey. If these virtual particles originate from an environment with a temperature below a threshold value indicated by $\hat{SST}$ (see Section 2 and Figure 1), the particle is assumed to originate close to Antarctica, and flagged as representing Antarctic‐endemic dinocyst species (see Section 2 and Figure 1 for an illustration). As such, model dinocyst endemism at the ocean bottom is determined by the percentage of virtual particles that started sinking in a surface environment with a temperature below $\hat{SST}$ (Figure 3).

Due to the circulation differences between eddying and noneddying simulations, the model‐derived occurrence of Antarctic‐endemic sedimentary dinocysts is clearly different between both configurations (Figure 3). While the endemism is more strongly dependent on latitude and a sharper boundary exists between low‐endemism and high‐endemism in LR4, sinking particles are transported further away from Antarctica in specific areas (especially near western boundary currents) in HR4. As a consequence, the occurrence of several recorded endemic species can be explained in HR4, while it cannot in LR4 (see e.g., site SanB). Moreover, the modeled endemism in the noneddying LR4 cannot match with both DSDP277 and MH at the same time, because these sites contain an opposite signal (i.e., MH contains endemic species and DSDP277 does not) while being located closely to each other. In HR4 on the other hand, the sedimentary particles in site DSDP277 (Figure 3) originate only from the warm waters of the southeastward flowing EAC, while the closely located site MH also contains particles originating from cold waters in the east, in agreement with the occurrence of endemic species at MH.

Overall, we find that the eddying model is able to produce a flow pattern which is consistent with plankton biogeographic patterns at all sites (Figures 3a and 3b). The model‐data fit improvement in HR4 compared to LR4 highlights the need for accurate reconstructions of the geographic boundary conditions (Baatsen et al., 2016) to optimize model‐data matches as in Figures 3a and 3b: It is the details in the ocean flow that induce a better model‐data fit in HR4 compared to LR4.

**Figure 4:**
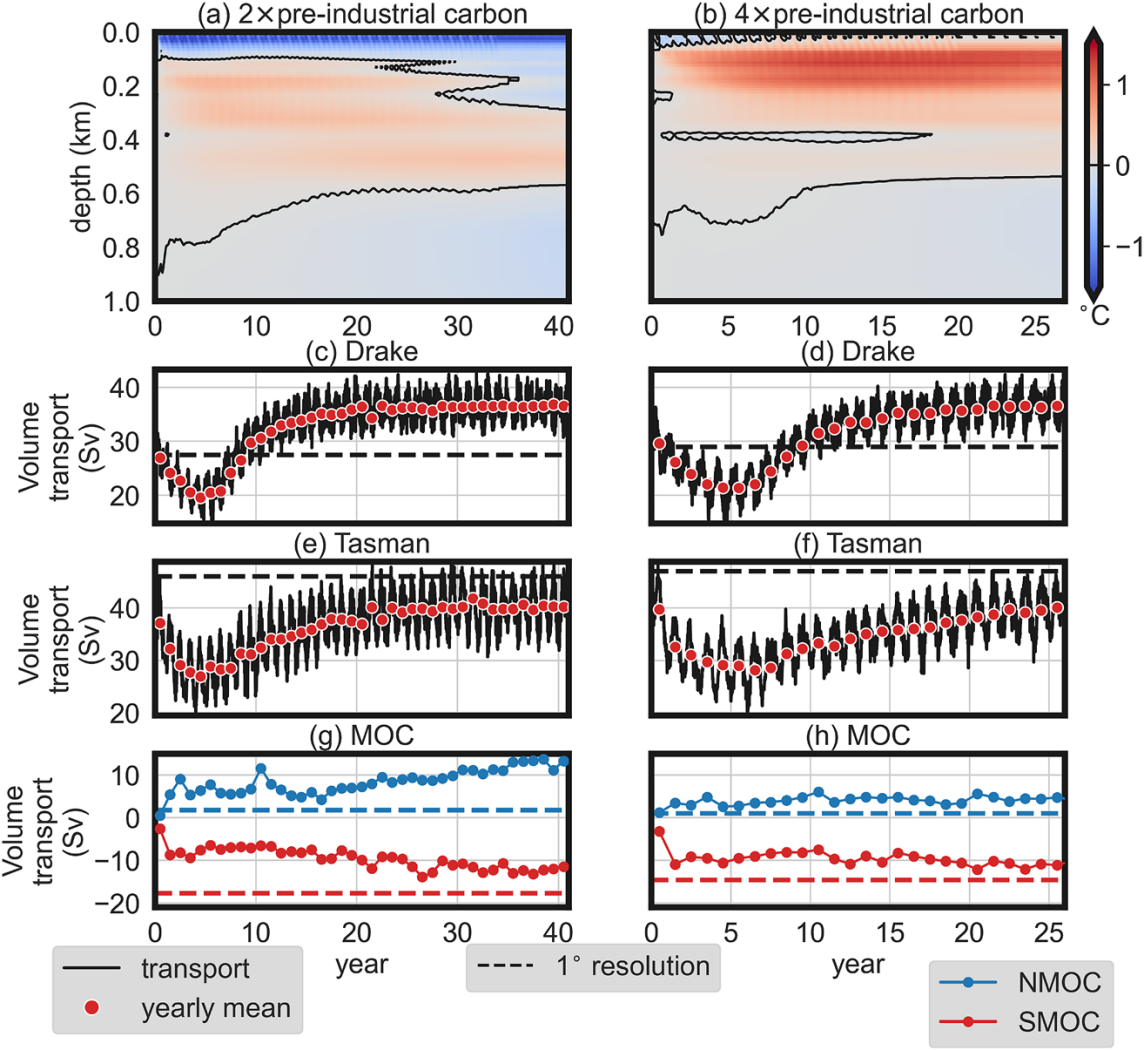
Response of the ocean model after initialization, HR2 (left) and HR4 (right). Note that the initial state of HR2 (HR4) corresponds to LR2 (LR4). (a, b) Depth‐dependent evolution of the horizontal mean temperature increase compared to the initialization state (upper 1 km only). Water volume transport through the (c, d) Drake Passage (65°W) and (e, f) Tasman Gateway (150°E). (g, h) Maximum of the northern and southern zonal mean meridional overturning. MOC, Meridional Overturning Circulation; NMOC, Northern MOC; SMOC, Southern MOC; Sv, Sverdrup.

The modeled dinocyst endemisms in the 2× and 4× preindustrial atmospheric CO_2_ configurations are similar (see Figures S2, S5, and S6 in Supporting Information S1), even though HR2 and HR4 are forced by a different atmosphere and respond differently after initialization (Figure 4). However, the transient response of the upper‐ocean equilibrates similarly in the 2× and 4× preindustrial CO_2_ cases in a few decades, which also results in a similar time‐mean surface flow (Figure S7 in Supporting Information S1). This implies that plankton biogeographic patterns and surface ocean circulation are to a large extent affected by bathymetry, rather than the climate boundary conditions (e.g., atmospheric CO_2_) of the model.

At the beginning of the HR2 and HR4 simulations, much of the energy input at the surface is used to set up the circulation and the development of eddies, as can be seen from a reduction of Southern Ocean gateway transports in the first 5 years (similar in both HR2 and HR4), after which they recover (Figures 3c–3f). The reduced Southern Ocean gateway transport is caused by the growing eddy field, which extracts potential energy from the stratification and reduces the isopycnal slopes (Figure S8 in Supporting Information S1). However, the gateway transports recover after 5 years. The Drake Passage transport (through the gateway between South America and Antarctica) exceeds the transport in the low‐resolution simulations after 9 years and equilibrates at a higher level. The increased Drake Passage transport could be caused by the lower (more realistic) viscosity that the high‐resolution models allow compared to the low‐resolution model (which becomes numerically unstable at this low viscosity value), while isopycnals do not necessarily become steeper compared to the noneddying simulations. Interestingly, the volume transport through the Tasman Gateway in HR2 and HR4 does not exceed the volume transport in LR2 and LR4. Instead, a larger fraction of the water is transported north of Australia, resulting in the stronger southeastward East Australian Current (EAC) in the South Pacific (Figure 2).

### 3.2 Model‐Data Comparison: SST

Now that the high‐resolution POP model simulates an Eocene Ocean flow, which is consistent with proxy data for ocean circulation, we compare the results of these simulations to proxy data for SST. SST distributions, however, are also influenced by the model background state and sensitive to their global‐scale equilibration. Moreover, the background flow affects the distribution of heat differently in the eddying versus noneddying simulations. Mesoscale eddies are important for the distribution of heat, and eddying ocean models do a better job in representing heat transport compared to noneddying models that use parameterizations for eddy‐induced heat transport (Dong et al., 2014; Griffies et al., 2015; Viebahn et al., 2016).

Indeed, heat change is distributed differently in the upper km of the eddying compared to the noneddying simulations (Figures 4a and 4b). Eddies efficiently transport heat to the subsurface (Delworth et al., 2012), which leads to subsurface warming in both eddying simulations (HR2 and HR4) and a lower vertical temperature gradient compared to LR2 and LR4. However, in HR2, the surface cools more, while the subsurface warms less compared to HR4.

Much of the heat transport change from LR to HR is related to the Southern and Northern Meridional Overturning Circulation (SMOC and NMOC, respectively), which has implications for the SST model‐data comparison. In both HR2 and HR4, North Pacific sinking develops (in a few decades) next to existing South Pacific sinking, while in the low‐resolution simulations there is only Southern Hemisphere sinking (see Figure S9 in Supporting Information S1). Overall, the North Pacific sinking leads to an increase in the NMOC and a decrease in the SMOC. These changes in the MOC are stronger in HR2 compared to HR4, and both the NMOC and SMOC are still increasing in magnitude at the end of the HR2 simulation.

The SMOC also differs in structure between the high‐resolution and low‐resolution simulations (see the mixed layer depth in Figure S9 in Supporting Information S1). In HR2 and HR4, more volume transport through Drake Passage increases the surface salinity in the South Atlantic resulting in denser surface water in the Weddell Sea (Toumoulin et al., 2020). Therefore, the main deepwater formation location is the South Atlantic in HR2 and HR4, while it is the South Pacific in LR2 and LR4.

These results imply that HR2 and HR4 are run long enough for the upper‐ocean circulation to equilibrate, while the deep ocean is not in equilibrium yet, as can be seen from the MOC in HR2 (Figure 4g). Although the transient evolution of the deep ocean circulation differs between HR2 and HR4, we can nevertheless investigate their impact on SST distributions and compare these to proxy data.

**Figure 5:**
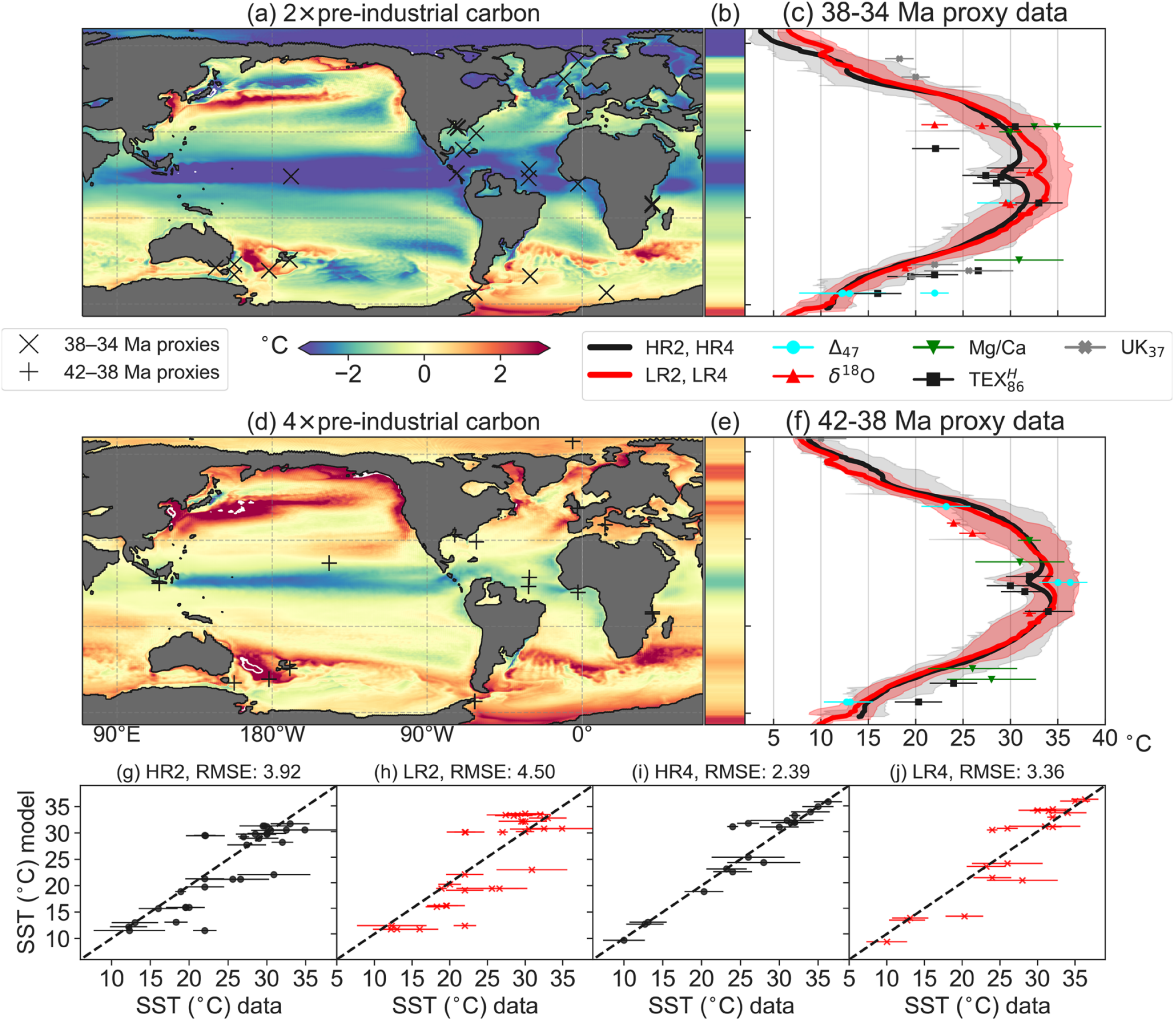
Model‐proxy data comparison: sea surface temperature (SST). The 2× and 4× preindustrial case are compared to SST proxy data of 38‐34 and 42‐38 Ma, respectively. (a, d) SST difference of the high‐resolution compared to the low‐resolution model with the site locations of the SST proxies for 2× and 4× preindustrial carbon configuration, respectively, and (b, e) their zonal mean. Solid white lines indicate ±5 °C contours. (c, (f) the zonally averaged annual mean SST in the high‐resolution (black) and the low‐resolution (red) model for 2× and 4× preindustrial carbon configuration, respectively. The shaded areas show zonal spread (i.e., minimum and maximum) of the annual mean SST. Markers indicate SST proxy estimates with their uncertainty. (g–j) Scatter plots between proxy‐derived and model‐derived SST for all four configurations, with root mean squared errors (RMSE). Error bars represent proxy calibration errors. To consider the paleolocation uncertainty of sites (van Hinsbergen et al., 2015), each site is compared to the model SST value from up to 3° distance of the site that minimizes the RMSE of the scatter plot (similar to Baatsen et al. (2020); see Figure S10 in Supporting Information S1 for a point‐to‐point comparison). The dashed black line is the one‐to‐one line representing the perfect match between model and proxy data.

Both the tropical and Arctic Ocean cool significantly in HR2 compared to LR2, while in HR4, the equatorial regions cool less and high‐latitude (north and south) regions warm more as compared to LR4 (see Figure 5). For both atmospheric CO_2_ levels, local SST differences between the high‐resolution and low‐resolution simulations mostly occur near western boundary currents of which the location shifts in the eddying simulation (Figures 5a and 5d). These shifts have an effect on the model‐data comparison at sites near western boundary currents. In fact, the EAC transports warm waters southeastwards in the southwest Pacific, which (partly) explains why sites in the southwest Pacific are found to be warmer compared to model simulations with a coarse resolution, which has been speculated to be a reason for the model‐data mismatch in the southwest Pacific before (Hollis et al., 2012). Notably, similar SST changes occur near the Kuroshio and Agulhas currents. The Weddell Sea warms up in HR2 and HR4 compared to LR2 and LR4, respectively, which is related to the South Atlantic sinking that occurs in HR2 and HR4.

Climate models generally do not produce the low meridional temperature gradients of warm climates as inferred from proxy data (Huber & Caballero, 2011; Sijp et al., 2014). While the simulations LR2 and LR4 were found to generate a lower meridional SST gradient compared to other models of 1° horizontal resolution or coarser (Baatsen et al., 2020), this gradient reduces further in HR2 and HR4. The tropics are cooler in HR2 and HR4 compared to LR2 and LR4, while in the zonal mean the southern high latitudes are only slightly warmer in HR4 (Figures 5d–5f). Regionally, there is, however, significant warming of Southern Ocean SSTs in HR4. Overall, this improves consistency between the high‐resolution model results and SST proxies in the tropics, while the modeled high‐latitude SST values are often still lower than the proxy‐derived SST values. The eddying simulations show stronger horizontal gradients in the time‐mean SST field compared to the noneddying simulations, which results in a higher time‐mean SST variation in the model around the sediment sample sites. The model‐data fit greatly improves in the eddying compared to noneddying simulations (Figures 5g–5j), although a mismatch with some sites remains (especially for the 2× preindustrial CO_2_ case) and the high‐latitude temperatures are overall lower compared to the proxy data.

Overall, the eddying ocean model improves the SST model‐data match from the noneddying model, because it alters the local transport of heat. However, the SST model‐data comparison is also sensitive to the model background state (i.e., the state of the ocean at a global scale), which depends on the used atmospheric forcing, paleogeography and long time scales phenomena, such as the deep meridional overturning circulation. Hence, the SST model‐data mismatch could be reduced even further if better model boundary conditions are used which lead to a more realistic background state of the late Eocene.

## 4 Conclusion and Outlook

We have shown that an eddying Eocene Ocean simulation provides a more detailed ocean flow compared to a noneddying version of the same model. As a result, model‐data mismatches in the geologic past (Baatsen et al., 2020; Bijl et al., 2011; Houben et al., 2019; Huber et al., 2004; Hutchinson et al., 2021; Lunt et al., 2021) can at least partly be explained by the lack of eddies in the ocean models used. Our eddying simulations of the late Eocene are better able to explain the occurrence or absence of endemic dinocyst species near Antarctica compared to noneddying simulations. The SST model‐data comparison also improved in the eddying compared to noneddying simulations.

The explicit representation of eddies in ocean models may have implications for comparison of models with other proxy types than considered here. For instance, pollen‐based temperature reconstructions imply that it did not freeze at the Antarctic coast during winter in the early Eocene (globally ∼6 °C warmer than the late Eocene), despite polar darkness (Pross et al., 2012). Eddy‐induced flow, and its impact on ocean heat transport, could in part explain such conditions.

The simulations in this paper are computationally expensive. However, other types of model setups may be interesting if computational capabilities are available. The strong influence of bathymetry on the eddying flow implies that the uncertainty of paleogeography reconstructions will have a major impact on model‐data comparisons. Future studies could make adaptations to the bathymetry within uncertainty of paleogeographic reconstructions, to find its impact on the modeled ocean circulation and model‐data comparison. These adaptations could also include more detail in the bathymetry, similar to Sauermilch et al. (2021).

Moreover, since the eddying flow has a direct response to bottom topography, it seems suitable for a downscaling, or eddy parameterization type of approach to obtain this influence of bathymetry on the flow with reduced computational costs. However, these type of approaches are found to be challenging in present‐day configurations (Fox‐Kemper et al., 2019; Lanzante et al., 2018; Nooteboom et al., 2020).

Second, we used the model equilibrium of the noneddying climate model simulations (which are in radiative equilibrium (Baatsen et al., 2020)) to start and force the eddying model. However, this switch induces a drift of the deep ocean circulation, which is not equilibrated yet in the high‐resolution simulations of this paper. Hence, the background state of the model will change further if the model is run for longer time periods (a few millennia). Future simulations may have the capabilities to perform longer simulations. These changes of the model background state on long time scales might have implications for the regional flow and the quality of the model‐data comparisons.

Finally, atmospheric feedbacks greatly influence the ocean model background state on long time scales, such as the meridional overturning circulation (Arzel et al., 2011; den Toom et al., 2012; Rahmstorf & Willebrand, 1995; Zhang et al., 2010). Hence, the high‐resolution ocean should be coupled to a high‐resolution atmosphere, which could further enhance the meridional transport of heat and lead to an improved model‐data comparison. So far, it has been shown that ocean‐atmosphere coupling in low‐resolution models does not necessarily lead to more poleward heat transport in an Eocene climate compared to the present‐day (Huber & Nof, 2006). However, coupling of the ocean with a high‐resolution atmosphere may result in relevant changes of the poleward heat transport, for instance due to resolving small‐scale features in the atmosphere such as tropical cyclones (Scoccimarro et al., 2011). An eddy‐permitting ocean could help facilitate a better overall match to SST observations in a fully coupled configuration, as it did in the present‐day (Delworth et al., 2012).

## Supporting information

Supporting Information S1

Movie S1

## Data Availability

The code used for this work and the results are distributed under the MIT license (Nooteboom, 2022a). The model data used to generate the main figures in this paper are publicly available on the Utrecht University Yoda platform (Nooteboom, 2022b).
