# Supplementary material for: Improved Model‐Data Agreement With Strongly Eddying Ocean Simulations in the Middle‐Late Eocene
Source: Paleoceanogr Paleoclimatol. 2022 Aug 17;37(8):e2021PA004405. doi: 10.1029/2021PA004405 (PMC9540656; doi:10.1029/2021PA004405)
Supplement: Supplementary file 1 — Supporting Information S1 [file PALO-37-0-s001.pdf]

Supporting Information for "Improved model-data agreement with strongly eddying ocean simulations in the middle-late Eocene"

Peter D. Nooteboom[1,2], Michiel Baatsen[1], Peter K. Bijl[3], Michael A. Kliphuis[1], Erik van Sebille[1,2], Appy Sluijs[3], Henk A. Dijkstra[1,2], and Anna S. von der Heydt[1,2]

[1]Institute for Marine and Atmospheric research Utrecht (IMAU), Department of Physics, Utrecht University, Utrecht

[2]Centre for Complex Systems Studies, Utrecht University, Utrecht, Netherlands

[3]Department of Earth Sciences, Utrecht University, Utrecht, Netherlands

Contents of this file

1. Figures S1 to S10

Additional Supporting Information (Files uploaded separately)

1. Caption for Movie S1

Introduction These Supplementary Materials include 10 figures and 1 animation that support the results described in the main article.

Movie S1. Animation of sea surface temperature during the spin-up of the HR2 simulation.

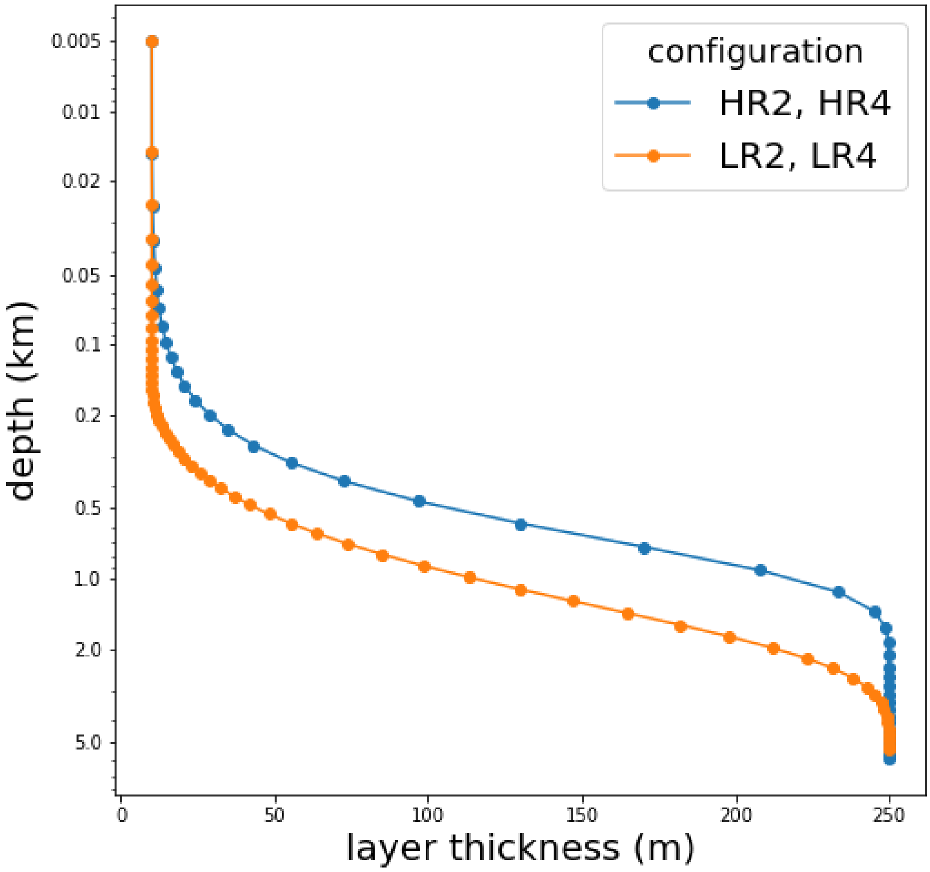

Figure S1. Layer thickness against depth (at T-points) for the high- and low-resolution configurations. The vertical resolution is almost the same for both configurations in the upper 100m. Notice that the vertical axis is logarithmic.

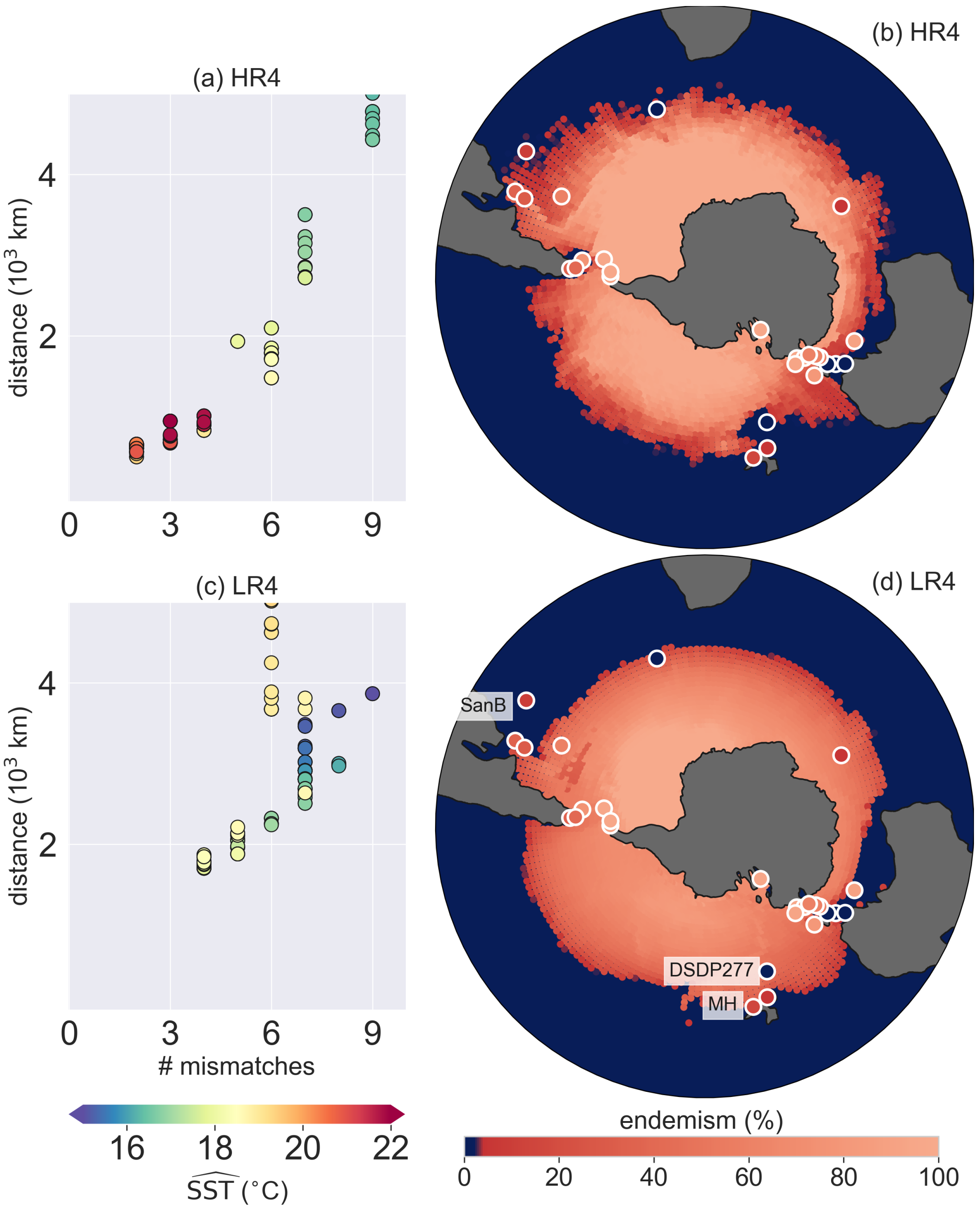

Figure S2. Same as figure 2, but with 25 m day^{-1} sinking speed.

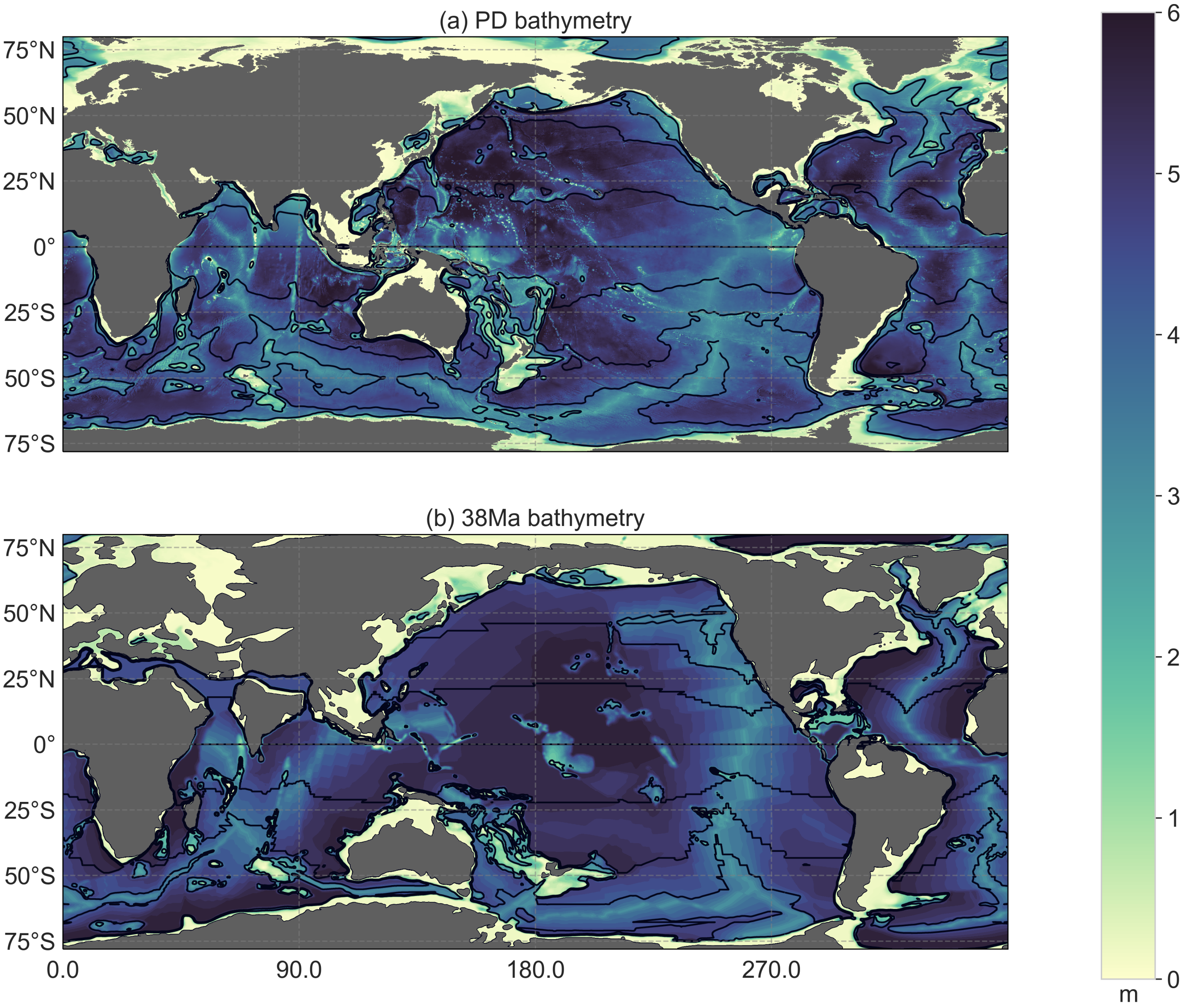

Figure S3. Global bathymetry in (a) the present-day (PD) and (b) the middle-late Eocene (38Ma). Black contours are lines of constant $\frac{f}{H}$ that the flow tends to follow in eddying simulations to conserve potential vorticity, with $f = 2\Omega\sin(\phi)$ the coriolis parameter (Ω is the rotation rate of the Earth and ϕ the latitude) and H the bathymetry.

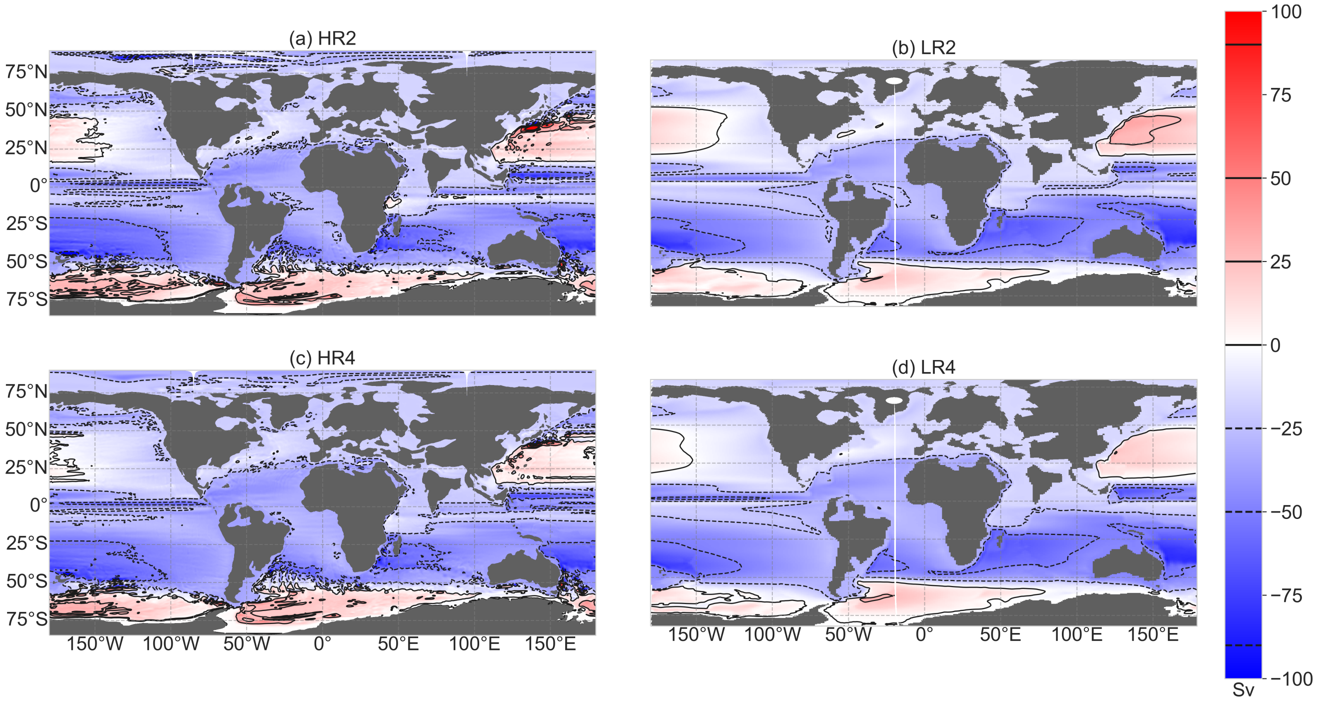

Figure S4. Barotropic stream functions of the annual mean circulation in all four configurations.

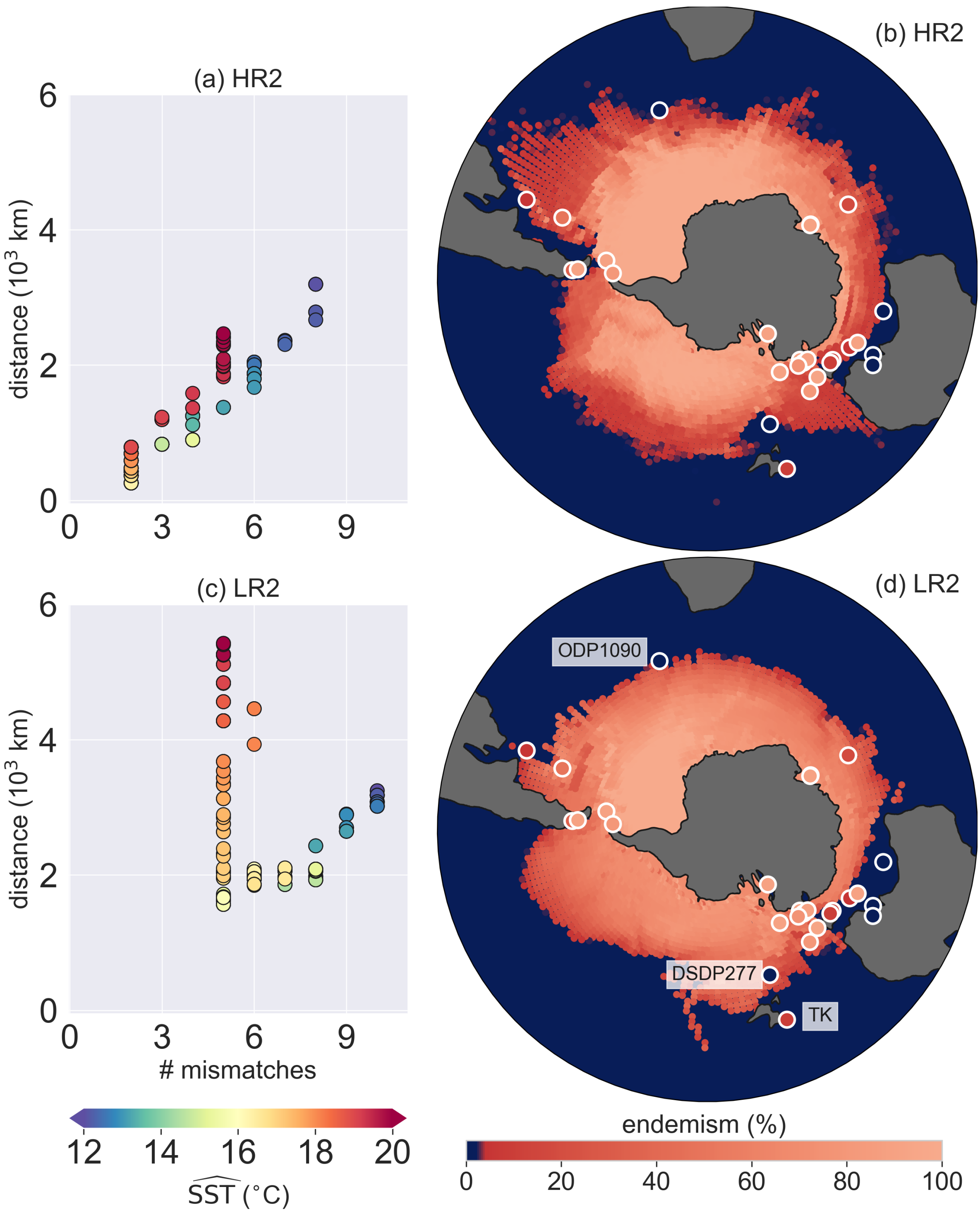

Figure S5. Same as figure 2, but for the 2×pre-industrial case (LR2 and HR2).

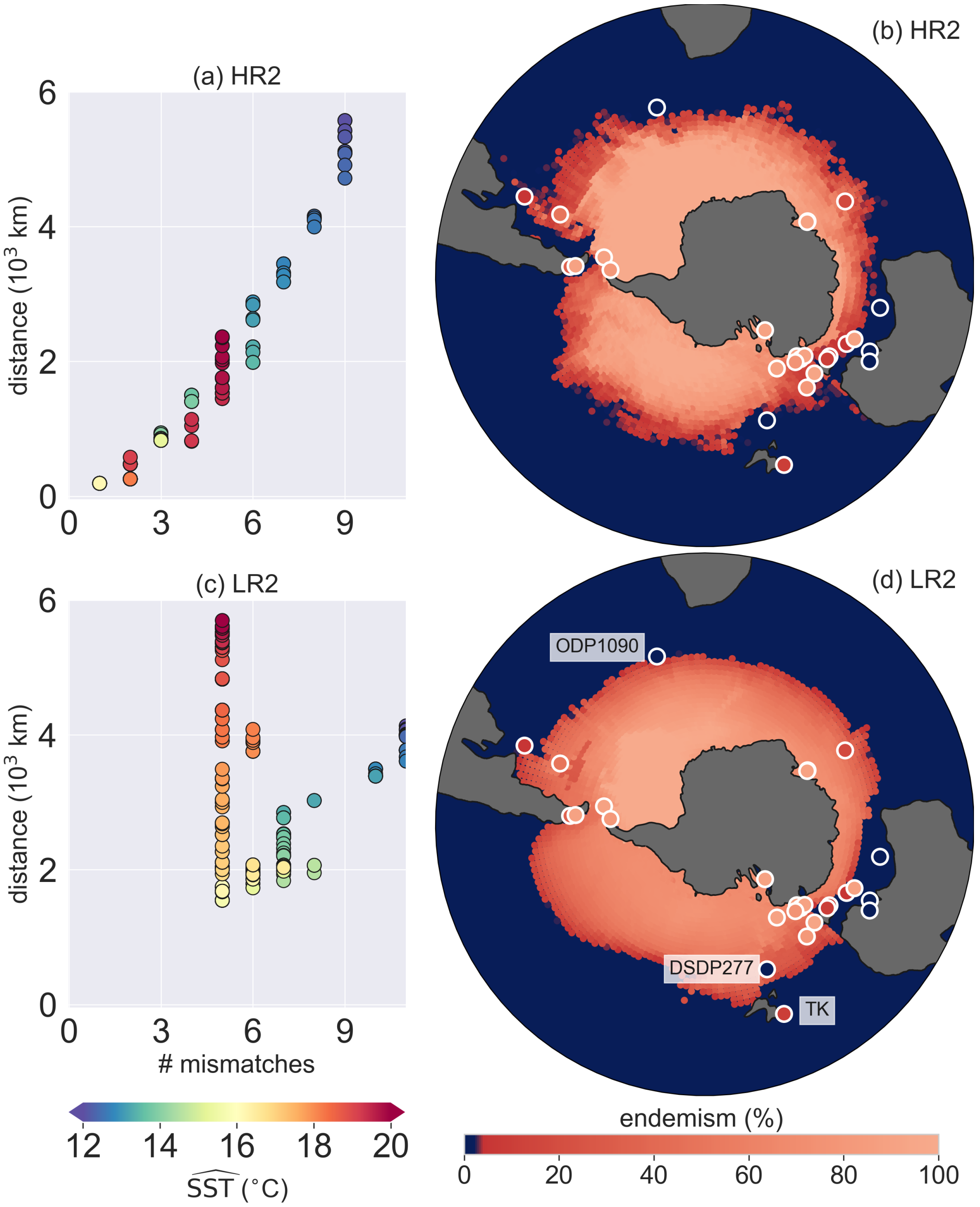

Figure S6. Same as figure 2, but with 25 m day^{-1} sinking speed and 2×pre-industrial case (LR2 and HR2).

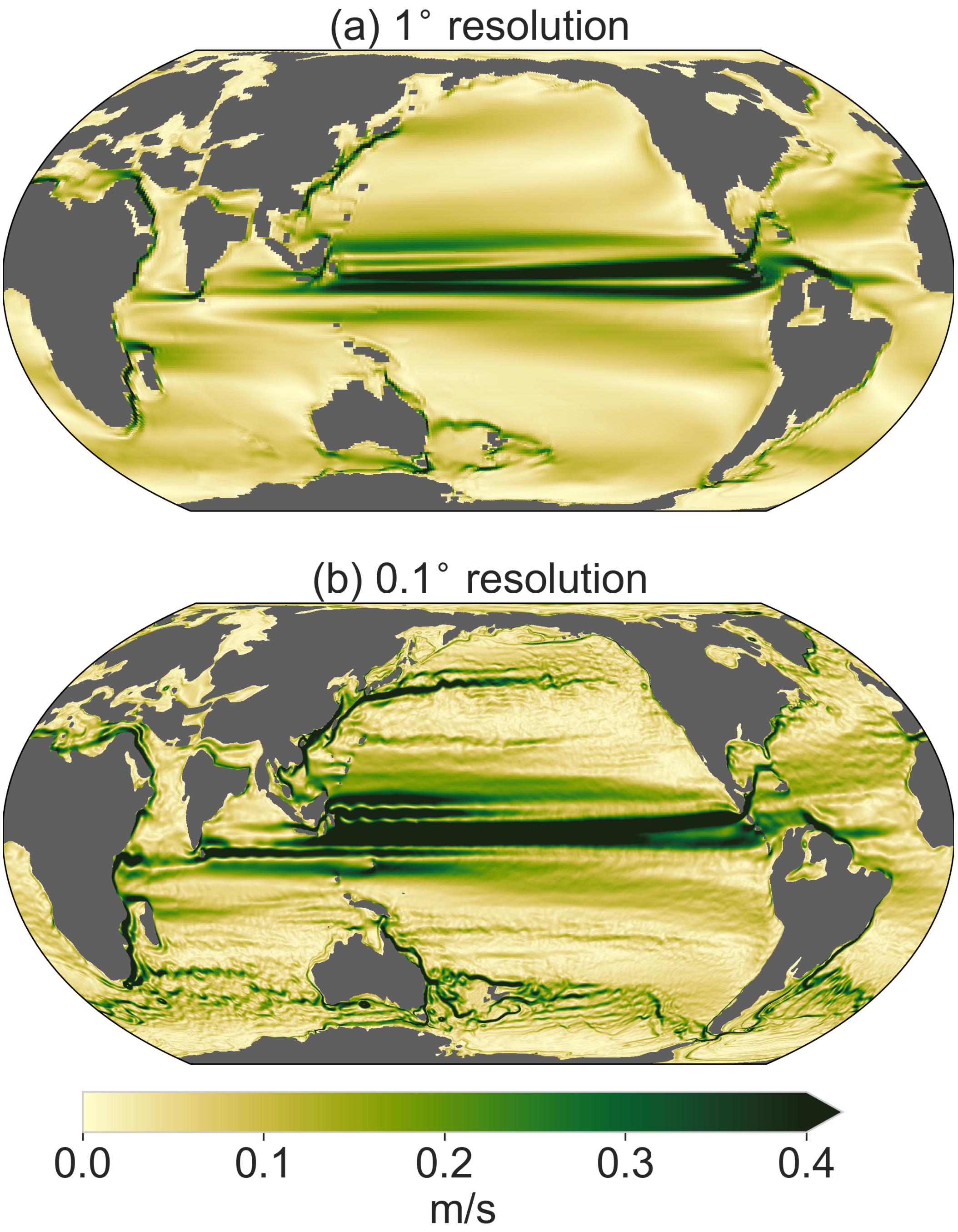

Figure S7. Same as figure 1, but for the 2×pre-industrial case (LR2 and HR2).

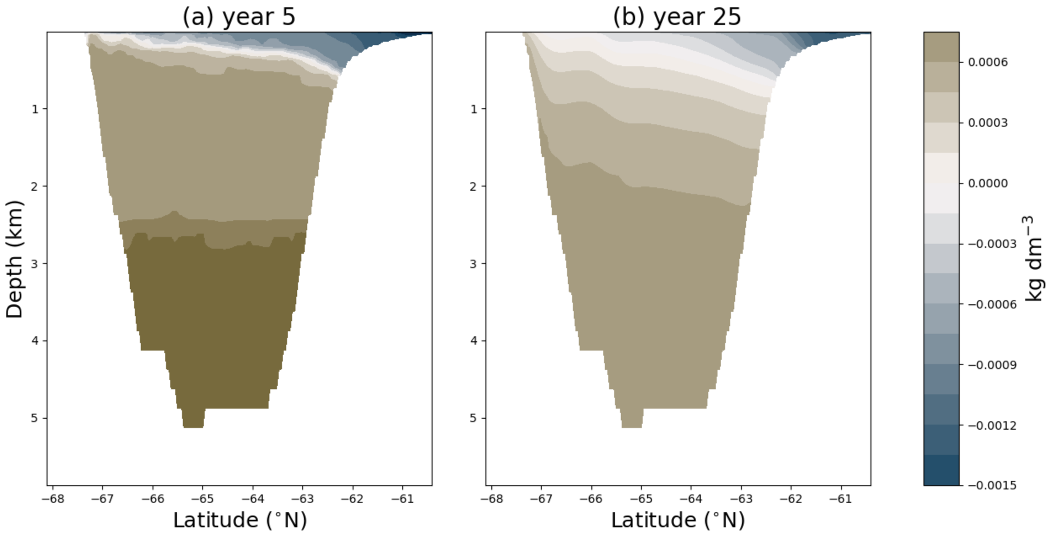

Figure S8. Annual mean potential density anomaly in year (a) 5 and (b) 25 in the Drake Passage (63.45°W) of the HR4 simulation.

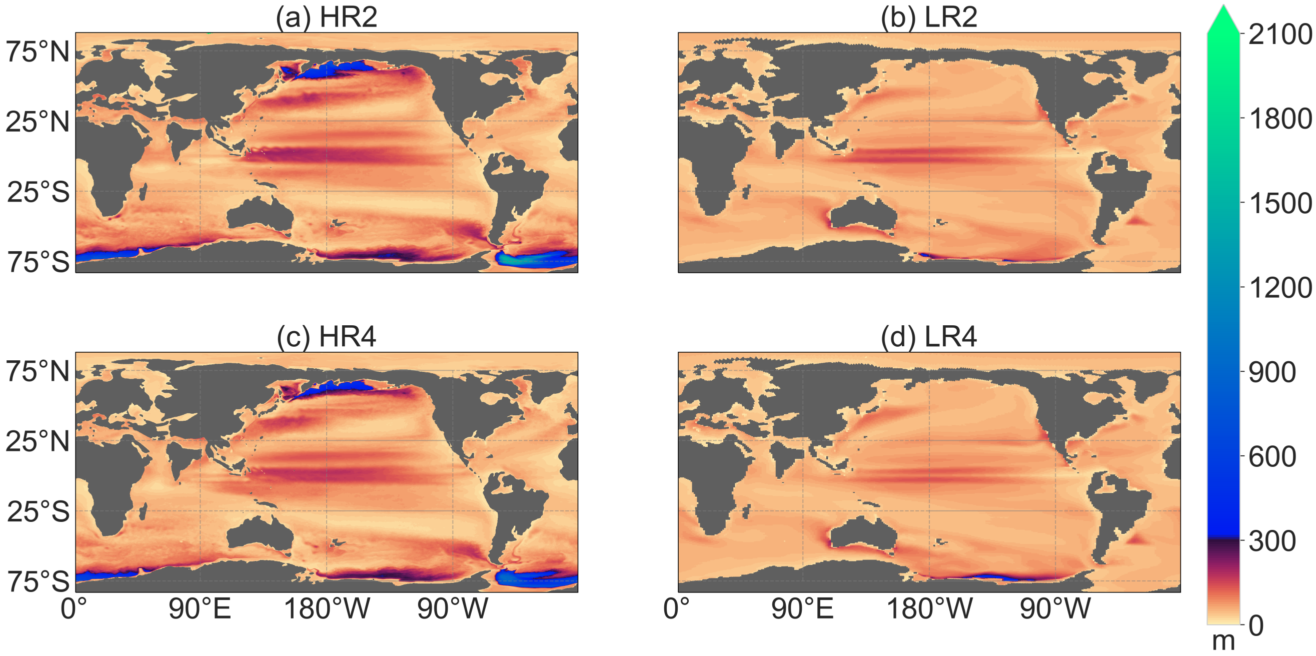

Figure S9. Maximum monthly mean of the mixed layer depth.

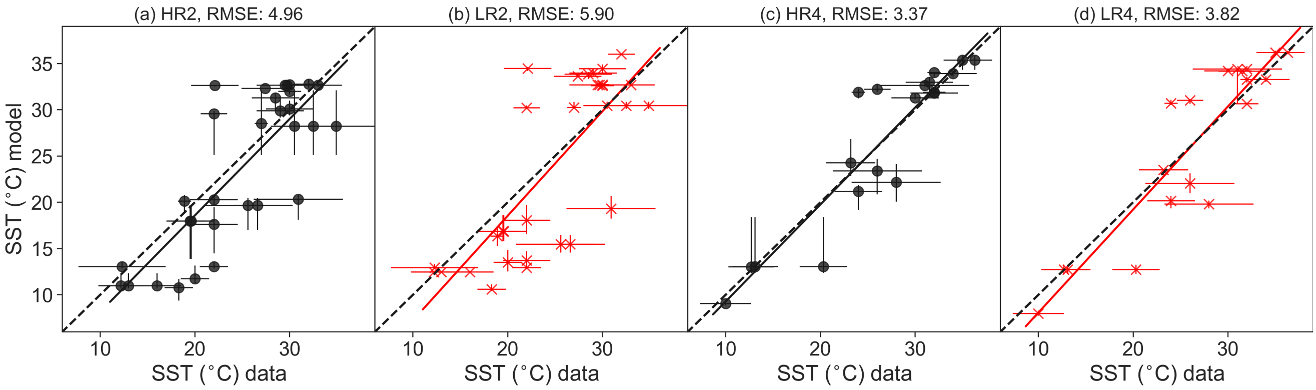

Figure S10. Same as figure 4g-j, but with a point-to-point comparison of model and data. The vertical uncertainty bars show the SST spread (minima and maxima) within a 4×4° box around the sites.
